# The Abuse of Hospitals

**Published:** 1890-01-18

**Authors:** John J. Weaver

**Affiliations:** House Surgeon, Southport Infirmary


### The Abuse of Hospitals.

By John J. Weaver, House Surgeon, Southport Infirmary.

Having spent almost the whole of the last four years in intimate association with the working of two provincial hospitals, I venture to give my experience of what is now rapidly becoming the burning question of the hospital world—viz., the abuse of hospitals. I regret to state at once that, personally, I have not a shadow of a doubt that our hospitals are extensively abused, and in that opinion I feel certain that the vast majority of hospital workers are agreed, but it is much more difficult to get the same approach to unanimity of opinion as to the actual form the abuse of hospitals takes. With the object of throwing some light on this side of the question, I shall attempt to point out as shortly as I can in what way, as far as my experience shows, hospitals are abused.

I will first take the case of in-patients. I believe, in provincial hospitals at all events, the abuse of hospitals by in-patients is practically absent, or if it exists at all it is so slight as not to be worth fighting about. I have never myself yet met with a case of an in-patient where, supposing the patient had not been treated in the hospital, I could see that any medical man could have benefited by the case. A case of illness, or accident of sufficient gravity, to be admitted into a hospital—probably a case of some month or so's duration and requiring constant daily attention and nursing—is hardly in my opinion a case where, even supposing the medical man had attended the patient at his own home, the remuneration the medical man might charge would in the end have repaid him for the trouble of his attendance.

But when we come to the out-patients, the case is very different. Here, I believe, hospitals are most extensively abused in three different ways—viz.: (1) In the case of minor accidents, (2) by out-patients who can well afford to pay, and (3) by patients who rightly should go to the parish.

To take each of these points separately. As far as I have followed the subject in the medical and other journals, the abuse of hospitals by minor accidents seems to me to have been very greatly overlooked. I may say, however, that in hundreds of cases even in my short experience of hospital work, I have attended patients as out-patients whose injuries were most trifling, and whose pecuniary circumstances were most certainly more than equal to paying a medical man for the attention they required. To take one case only. Quite recently I attended the head engineer of a timber yard for a simple cut by a chisel across one knuckle. The wound required one stitch, and had healed by the second visit. For this same patient I also had to sign (of course gratuitously) a certificate by which he obtained sick pay from a club for a few days. This patient, I do not hesitate to say, could well have afforded payment for the attention he required to any practitioner, and I only give this one case as an instance of hundreds of similar cases that constantly attend our hospitals, not so much in the town I am in at present (where accidents are comparatively rare), but in the manufacturing districts. In one hospital in a manufacturing district in which I was house surgeon, it was quite possible to tell what firm of employers a patient came from by the trivial nature of the injuries for which the men attended, and these men were in receipt of capital wages.

To take the second point. The abuse of hospitals by those who can well afford either to pay a doctor or to join a medical club is, I believe, well recognised, and I would only point out that in my opinion this abuse is now rapidly on the increase, on account of the greatly increasing faith the public generally are getting in hospitals. In my experience there are few men who, thinking they can benefit the health of their child, for instance, by getting gratuitous medical advice at a hospital, will hesitate as to whether or not they ought conscientiously to do so.

To take the third point—viz., the use or abuse of hospitals by people who on account of their extreme poverty ought properly to apply to the parish for relief. These people often want food quite as much as medicine, but on account of the greatly superior medical attention they receive at the hospitals, they prefer to go to the hospitals even though in the matter of food they are half starving. I myself often attend patients who a week or so back were in the workhouse, or under the parish doctor, but who, being dissatisfied perhaps, or from other reasons, come to the hospital. As long as these people are not actually in receipt of parish relief at the time they come to the hospital they are eligible for treatment there. It is a fact, therefore, that in the treatment of these cases the work of voluntary hospitals and the Poor Law medical department very much overlaps, and that therefore the charitable public (through their subscriptions to hospitals and their payment of rates) are, in so far as the treatment of these cases is concerned, paying twice over for the same thing.

The Poor Law medical officers are keenly alive to this side of the question, and as they are themselves generally very poorly paid for their services, it is distinctly to their interest, by refusing material relief or offering these people under all circumstances the workhouse, to drive them off their own hands on to the hospitals. I am at the present time attending a poor girl who already once at my request, on account of her poverty and the non-suitability of her case for admission into the hospital, went into the workhouse, but who now absolutely refuses to go back to the parish. She prefers to get her medicine from the hospital, though I know in the matter of food she is half starving. There is one other side of hospital work which I think is objectionable in principle, and that is home visiting. This is, in my experience, purely and simply a case of using the money of the charitably disposed to pay towards doing the work the Poor Law medical department is intended to do.

We come, lastly, to the question of remedies, and on this point I will only offer a few suggestions as to how possibly some of the present abuses of hospitals may be remedied.

First, with regard to the case of minor accidents; and this, I believe, is one of the most difficult forms of abuse to deal with. It is clearly impossible to leave to the public to decide whether an accident at the time of its receipt is a simple one, which may be attended by any practitioner, or one which should go straight away to the hospital. Possibly a system of enquiry (after the Manchester Infirmary plan) into all these cases after they have been treated at the hospital might be adopted, and payment insisted on where abuse was found—payment not advocated in the interest of the hospital, but simply as a preventive of abuse, by lessening the inducements to go to the hospital. The scale of charges and other details would probably be best settled on local considerations.

In the case of out-patients (not accidents) who can afford to pay for their treatment, what can be done? They should certainly, in my opinion, be prevented going to the hospitals. I am acquainted with one institution in which this abuse is met by having a small sub-committee, sitting each day half an hour before the medical officers attend to see the patients, and this committee investigates, verbally at least, into the circumstances of each new patient seeking treatment. This method is, I believe, effective but perhaps where there are a large number of patients is rather laborious. The very fact, however, of there being such a committee for patients or their friends to meet prevents many undeserving cases presenting themselves, and the deserving patients among the working classes less resent an enquiry into their circumstances by such a committee than they do an enquiry by a paid agent. To meet the case of patients who, on account of extreme poverty, properly belong to the parish, it is very difficult to suggest any remedy, except that of improving the attention they are in the habit of getting from the parish, by insisting on the parish authorities doing their work efficiently. It is a frequent experience of mine to find these people endure semi-starvation rather than give up their medical treatment at the hospital.

Any great reform of the present hospital out-patient work should deal thoroughly with the question as to how far down in the social scale the hospital system is intended to go; and whether, except in incurable and other extreme cases, the hospital system should, or should not, be allowed to entirely supersede the outdoor medical relief of the Poor Law.

